# The recognition pathway: reconceiving inventorship and obviousness for iterative CRISPR-AI convergence

**DOI:** 10.1093/jlb/lsag028

**Published:** 2026-11-10

**Authors:** Hassan Abdulhakim Bahebe, Li Xiaoming

**Affiliations:** School of Law, Xi’an Jiaotong University, Xi’an, Shaanxi, China; School of Law, Xi’an Jiaotong University, Xi’an, Shaanxi, China

**Keywords:** artificial intelligence, biotechnology, CRISPR, inventorship, patent law, PHOSITA

## Abstract

Clustered Regularly Interspaced Short Palindromic Repeats -Artificial Intelligence (CRISPR-AI) convergence generate therapeutic candidates via iterative human-machine workflows that disrupt the inventorship criterion of patent law based on conception. In CRISPR-AI studies, AI systems suggest thousands of guide RNAs or Cas variants; human researchers select, confirm, and optimize them via experimental feedback loops that conceal the locus of conception. The existing patent doctrine, which demands a clear and final concept in the human mind, fails to align with inventions in which AI comes up with solutions that human researchers had not anticipated. This Article advances a *recognition pathway* for CRISPR-AI inventorship that maintains the natural-person baseline while attributing inventorship to humans who exercise *claim-defining technical judgment* in identifying patentable value within AI outputs. The Article reconceives the person having ordinary skill in the art standard for AI-enhanced biotechnology, rejecting both inflexible pre-AI baselines and the 'computer having ordinary skill in the art' proposal. A five-factor test is employed to assess AI-generated CRISPR inventions on non-obviousness in terms of problem structure, predictability of solutions, and quality of human judgment. The framework provides a computational biotechnology human-accountability grammar that is administrable, but not legally granted to machines.

## I. INTRODUCTION

In April 2024, Profluent Bio announced OpenCRISPR-1, the first open-source gene editor fully trained on artificial intelligence,^1^ with peer-reviewed publication following in July 2025.^2^ The system was developed using large language models that were trained on more than 26 terabases of prokaryotic and phage genome sequences, producing a Cas9-like protein with 403 amino acid differences from the canonical SpCas9, none of which are found in nature.^3^ Human cell experimental validation showed similar efficiency in editing to natural CRISPR systems, and a 95 per cent reduction in off-target effects.^4^ The AI had not simply optimized an existing tool; it had written a new biological system that was subsequently chosen, synthesized, and validated by human researchers.^5^ This raises a fundamental question: who qualifies as the inventor under patent law?

This issue has become increasingly significant in contemporary legal discourse. In 2012, the adaptation of CRISPR-Cas9-based systems to genome editing made the accuracy of DNA editing available in medicine, agriculture, and synthetic biology.^6^ Simultaneously, artificial intelligence has evolved from analytical tool to generative co-creator. AlphaFold 3 can now predict protein-nucleic acid-small molecule interactions with experimental accuracy,^7^ while CRISPR-GPT is an orchestration of large language model agents that are experimentally validated in closed loops to automate gene-editing research.^8^ The CRISPR-AI convergence yields therapeutic candidates, engineered crop characteristics, and synthetic biological systems via iterative workflows where AI generates unexpected solutions, which humans discover, test, and optimize.^9^

Patent law remains tied to a conception-based theory of invention. Under US patent doctrine, inventorship must consist of the conception in the mind of the inventor of a clear and fixed idea of the entire and working invention.^10^ The 2025 United States Patent and Trademark Office (USPTO) Revised Inventorship Guidance reiterates this criterion, believing that AI-assisted inventions can be patented only when one or more natural persons are involved in conception, and that there should be no special doctrinal consideration of machine participation.^11^ The major forums have consistently disqualified AI systems as inventors: the UK Supreme Court, the Legal Board of Appeals of the European Patent Office, the Federal Circuit, and most recently the Swiss Federal Administrative Court and the Intellectual Property High Court of Japan have all found that only natural persons should be considered inventors.^12^

Nonetheless the conception criterion finds it difficult to deal with the unique dynamics of CRISPR-AI. Consider the following illustrative workflow.^13^ A research team uses CRISPR-GPT to design a high-fidelity Cas9 variant to treat sickle cell disease with fewer off-target effects. The system, which is trained on curated sequence-function data, suggests hundreds of variants. The principal investigator establishes rejection levels according to the anticipated specificity, picks three candidates to be experimentally validated, and repeats: experimental results update the model, which produces refined proposals. The team finds a variant after six cycles that has unforeseen Protospacer Adjacent Motif (PAM) specificity that no human had imagined.^14^ This raises the central question: who is the inventor in this scenario?

Current frameworks provide limited guidance for CRISPR-AI inventorship. The human-machine division of labor is indistinct at the point of conception when AI is treated as a tool.^15^ The acknowledgment of AI as an inventor contradicts established statutory obligations and poses insurmountable legal responsibility questions.^16^ Recent research has suggested augmented inventorship, which maintains human inventorship but renders the role of AI visible by directability (ex-ante steering) and traceability (audit trails).^17^ This approach takes into account key aspects of human–AI partnership and does not easily involve the iterative feedback of CRISPR-AI where experimental validation and model refining generate a unique cognitive response, the view that there is patentable value in AI-generated products.

This Article advances a *recognition pathway* for CRISPR-AI inventorship. The thesis is that patent law must maintain the natural-person standard while recognizing a distinct cognitive contribution: *claim-defining technical judgment* exercised by humans who (i) identify patentable value in AI outputs that they did not and could not have generated independently; (ii) validate that value through experimental demonstration; and (iii) integrate those outputs into a coherent inventive concept through iterative feedback. Recognition is not mere appreciation of results, routine screening, or post hoc ratification. It requires active intellectual engagement in which the human researcher applies domain-specific expertise to determine which AI-generated variants warrant experimental investment, distinguishes genuine technical effects from spurious correlations or model hallucinations, and recognizes unexpected properties such as novel PAM specificities or off-target profiles that fall outside the anticipated solution space.

The Recognition Pathway rests on two complementary design criteria. First, recognition captures claim-defining technical judgment through which researchers identify AI-generated outputs warranting experimental validation and iterative refinement. Second, iterative integration recognizes the continuous feedback between computational prediction and experimental validation. Together, these criteria underpin the proposed conception-recognition-integration model of inventorship and the reinterpreted PHOSITA standard developed in Part IV.

These design criteria are particularly important in CRISPR-AI because experimental validation is indispensable for determining whether AI-generated outputs possess genuine technical effects rather than computational artifacts. CRISPR-AI thus serves as the archetypal case study for a broader class of experimental-validation-intensive AI inventions encompassing drug discovery, materials science, and synthetic biology where biological or physical unpredictability necessitates human judgment to transform computational predictions into patentable inventions. Building on the proposed inventorship framework, this Article develops *a five-factor obviousness test* that makes AI capabilities legible as predictability indicators while preserving human judgment as the evaluative baseline. This test fills a critical gap left by the 2025 USPTO Guidance, which acknowledged that 'similar questions... may arise under 35 U.S.C. § 103 but offered no analytical framework for addressing them.

The framework evaluates problem structure, solution predictability, and the quality of human technical judgment, thereby addressing a significant gap in existing patent doctrine.^18^ The discussion draws on recent jurisprudence in the United States, Switzerland, Japan, and China to show that jurisdictional fragmentation undermines incentives to innovate and suggests harmonized standards via World Intellectual Property Organization (WIPO) coordination.

The remainder of the Article proceeds as follows. Part II explains the convergence of CRISPR-AI, with a focus on the iterative feedback loops that help to differentiate it not only with traditional biotechnology but also with generic AI-assisted research. Part III develops the recognition pathway doctrine as an alternative to current methods and situates it within the theoretical foundations of patent law. Part IV reinterprets the PHOSITA standard in relation to CRISPR-AI, which offers the five-factor obviousness test. Part V examines comparative jurisdictional strategies and suggests harmonization strategies. Part VI deals with access and ethical implications. Part VII responds to the anticipated objections. Part VIII concludes.

## II. THE CRISPR-AI CONVERGENCE

A combination of CRISPR-Cas9 gene editing and generative artificial intelligence has arrived, creating a unique research paradigm: iterative human-machine workflows where experimental data are recursively used to inform computational prediction. This Part examines CRISPR-AI convergence, focusing on the iterative control loops that distinguish it from both traditional biotechnology and conventional AI-assisted research.

### I‌I.A. From Bacterial Immunity to Genome Engineering

CRISPR (Clustered Regularly Interspaced Short Palindromic Repeats) technology is based on adaptive immunity in bacteria.^19^ The CRISPR-Cas9 system guides the Cas9 nuclease via a guide RNA (gRNA) to specific sequences of DNA, allowing gene knockout, insertion, or repair using precise double-strand breaks.^20^ After it was demonstrated that genome editing was programmable in 2012,^21^ CRISPR has demystified genetic manipulation, lowering the cost and technical access in the medical, agricultural, and industrial applications landscapes.^22^

One prominent example is therapeutic application. The United States Food and and Drug Administration (FDA) approved the first CRISPR-based therapy for sickle cell disease in 2024,^23^ and clinical trials are underway for cancer, blindness, and rare genetic diseases. Examples of agricultural uses are crops that are nutritionally enhanced and resistant to water erosion, which may not be subject to Genetically Modified Organism (GMO) rules since they do not include alien DNA.^24^ Industrial uses include the sustainable production of chemicals as well as bioremediation.^25^

The patent landscape reflects this commercial significance. As of 2025, foundational patent rights have been a subject of dispute between the University of California, University of Vienna and Emmanuelle Charpentier (CVC) group and the Broad Institute with multi-jurisdictional eukaryotic use cases.^26^ In May 2025, the Federal Circuit vacated and remanded a Patent Trial and Appeal Board (PTAB) decision that had awarded priority for eukaryotic CRISPR/Cas9 to the Broad Institute, finding that the PTAB had improperly mixed the legal standards of conception and reduction to practice.^27^ This created uncertainty for licensing and poses risks to AI-enhanced CRISPR systems.

### I‌I.B. AI Integration: From Analysis to Creation

Artificial intelligence has evolved from an analytical tool into a co-creator of biotechnology. In 2020, AlphaFold 2 solved a 50-year-old biological problem by predicting protein structures with experimental accuracy.^28^ These advances were extended by AlphaFold 3 in 2024 to protein–nucleic acid and protein–small molecule interactions.^29^ In more than 1000-fold the known protein universe using conventional techniques, DeepMind has generated structural predictions for more than 300 million proteins.^30^

This computational capability raises important questions for patent disclosure doctrine. More specifically, an important question arises: where the applicant relies on AI-predicted protein structures rather than experimentally determined structures, does the specification enable a person of ordinary skill in the art to practice the invention without undue experimentation? The dilemma of CRISPR-AI is urgent: AI programs now create new base editors and prime editors–sophisticated CRISPR systems with single-nucleotide edits with no double-strand breaks. Beam Therapeutics has developed base-editing Therapy for alpha-1 antitrypsin deficiency and sickle cell disease clinical trials.^31^

The role of AI depends on how it is used. In some applications, AI functions as a search engine that narrows sequence space to identify optimal CRISPR guides.^32^ In other scenarios, generative models create genuinely novel protein structures based on the training with evolutionary data. The line between ‘AI-assisted’ and ‘AI-generated’ inventions is critical when AI proposes CRISPR variants that human scientists never anticipated discovering, a situation that is increasingly common with state-of-the-art generative models.

### I‌I.C. The Convergence: Iterative CRISPR-AI Workflows

Through the convergence of CRISPR and AI, researchers develop iterative workflows that challenge the traditional human–machine dichotomy underlying current patent doctrine.

The iterative workflow described in the Introduction, where a research team employs CRISPR-GPT to design and experimentally test Cas9 variants in sickle cell disease therapy, has three features that set CRISPR-AI apart from both traditional biotechnology and generic AI-assisted invention. First, generative autonomy: the AI produces solutions beyond the reach of human anticipation. Second, experimental grounding: AI performance is validated by human judgment, through physical demonstration, not through pure computational assessment. Third, recursive refinement: generative models are refined through co-evolution driven by experimental feedback.^33^ These features are described in detail in Part III as the basis of the recognition pathway.

In 2025, the Swiss Federal Administrative Court recognized this problem by deciding that a human who contributes significantly to the processing of AI data, regards the AI-output as a patentable invention, and applies for patent protection may be considered an inventor. But the Court did not explain how this standard of ‘recognition and appreciation’ should operate in complex, iterative research environments the very context in which CRISPR-AI workflows emerge. The 2025 USPTO Revised Inventorship Guidance, by contrast, reiterates the principle that conception must be in the ‘mind of the inventor’ regardless of AI use, and leaves open the doctrinal question of how to evaluate inventorship when inventive insight is generated through iterative human–AI feedback loops.

### I‌I.D. Analytical Categories

This Article employs three analytically distinct categories. AI-generated invention refers to inventions in which AI systems generate technical solutions without human contribution to the specific solution and therefore remain unpatentable under Thaler. AI-assisted invention involves human-controlled AI tools in which the human conceives the solution and AI assists its implementation. CRISPR-AI hybrid invention, the focus of this Article, involves iterative co-creation in which AI generates unforeseen technical solutions that humans discover, refine, and reduce to practice.^34^

The distinction is doctrinally significant. Where AI systems independently generate CRISPR systems with novel PAM specificities or enhanced fidelity, traditional inventorship analysis struggles to identify human conception. Human researchers provide training data and define research objectives, while the AI system generates the resulting technical solution. Under the current USPTO framework, inventorship turns on contribution to conception regardless of AI use. However, where AI generates an unforeseen technical solution, the attribution of conception becomes doctrinally uncertain.

The defining feature of this framework is its co-evolutionary development loop, in which experimental validation informs computational prediction, which in turn guides further experimentation. Unlike conventional AI-assisted invention, where AI primarily implements human-directed solutions, CRISPR-AI is characterized by iterative experimentation and computational refinement.

This dynamic calls for a more nuanced framework for attributing inventorship, one that recognizes meaningful human intellectual contributions across the iterative research process. Under this framework, the researcher does not need to independently conceive the exact technical solution that is ultimately claimed, but instead frames the scientific problem, sets the experimental parameters, interprets unforeseen findings, validates results, and exercises scientific judgment.

The Recognition Pathway is not intended to establish a field-specific rule for CRISPR-AI inventions. Rather, CRISPR-AI serves as the principal case study because its iterative interaction between computational prediction and experimental validation exposes the limitations of conventional inventorship doctrine particularly clearly. The proposed framework remains technology-neutral and may extend to other fields in which AI-generated technical outputs acquire inventive significance through subsequent human evaluation, refinement, and scientific judgment. *In this respect, CRISPR-AI functions as the archetype for experimental-validation-intensive AI inventions across biotechnology, materials science, and synthetic biology, not as a sui generis exception to technology-neutral patent doctrine.*

## III. THE RECOGNITION PATHWAY DOCTRINE

The inventorship criterion of patent law developed around the concept of conception does not support the CRISPR-AI iterative feedback-loop.^35^ The existing patent legislation lacks a clear definition of the attribution of conception in the case of invention by distributed human–AI systems, which poses an immediate doctrinal uncertainty. Where AI generates unforeseen solutions that humans identify, validate, and refine, the locus of invention is not conception in the traditional sense but recognition of patentable value in machine output. This Part develops the Recognition Pathway as a distinct doctrinal framework, distinguishes it from existing approaches, and situates it within the theoretical commitments of patent law.

### I‌II.A. The Limits of Existing Frameworks

The existing methods of AI inventorship have three poles. The AI-as-tool perspective considers generative systems as complex tools, maintaining the conventional human inventorship and leaving the analysis of evidence intact.^36^ Its strength is administrative simplicity; its weakness is evidentiary opaqueness. It refuses to mandate the structured documentation of the interaction of human judgment with AI outputs during conception, which denies examiners and courts the resources to assess inventorship in iterative processes.^37^

The AI-as-inventor methodology calls for the acknowledgment of AI systems as inventors where machine-generated outputs meet claim-defining characteristics.^38^ Descriptive candor is pushed to the extreme of the law where, in the most prominent forums, only natural persons are accepted by current statutes and case law. The collateral consequences are substantial: who would execute the declaration, bear duties of disclosure, assign title, or answer for misrepresentation? Absent comprehensive statutory overhaul, this path is foreclosed.^39^

In between these poles is augmented inventorship, which maintains the human-inventor baseline but makes the generative role of AI visible by directability (ex-ante steering) and traceability (audit trails). Directability models independent human intellectual judgment used to control model behavior; traceability needs records that are reviewable, relating human reasons to claim elements. This method is a step forward of AI-as-tool as it involves documentation that is organized, but it does not cover the unique dynamics of CRISPR-AI.

The limitation is temporal. Directability focuses on ex ante framing, problem definition, threshold setting, data curation, prior to the start of generative search. But the iterative workflows of CRISPR-AI entail ex post recognition: experimental validation informs us which variants created by the AI have startling technical implications, which are then used in the generative models.^40^ It is not just human control to drive search parameters but determining which among thousands of outputs generated are worthy enough of additional investment, a cognitive process unique to traditional conception and augmented directability.^41^

### I‌II.B. The Recognition Pathway: A Distinct Doctrinal Framework

The recognition pathway acknowledges human inventorship in a situation in which researchers use intellectual judgment to discover, confirm, and transform into practice AI-generated solutions that were and could not have been discovered and identified in the traditional way. The framework is organized into two design criteria.

Recognition is the practice of claim-defining technical judgment by a natural person who, when presented with an AI-generated output that is not within the human-anticipated solution space, (a) recognizes that the output has patentable utility, novelty, or non-obviousness; (b) confirms such recognition by experimental demonstration of reproducible technical effects; and (c) incorporates the output into a definite and permanent conception of the claimed invention. Recognition meets the Pannu standard of significant contribution to conception since it converts an AI-generated possibility into a legally cognizable invention by human intellectual effort that is contemporaneously recorded and claim-specific.

Recognition differs fundamentally from the mere selection or post hoc ratification of AI-generated outputs. Rather, it requires more than blind, algorithmic, or random selection. Technical effect judgment is needed to operate recognition where one must be able to assess whether an AI-generated variant produces the desired therapeutic effect, whether the generated variant interacts with the desired target or cleaves the target, whether experimental observations demonstrate that the generative model made predictions that were not made by the experimental results.^42^ This is based on the knowledge in the domain, ie, molecular biology, pharmacology, clinical translation, which AI systems lack.

Iterative Integration refers to the recursive feedback of CRISPR-AI workflows, where experimental outcomes are used to recursively predict computationally, generating new candidates to be experimentally tested in the next cycle. In contrast to linear invention, in which conception is followed by reduction to practice in discrete temporal steps, iterative integration allocates cognitive work between human–AI feedback loops.

In these workflows, no individual human has a definite and permanent conception of the full and functioning invention in advance; rather, conception solidifies through experimental confirmation that establishes which AI-generated outputs have unanticipated technical qualities. Iterative integration is not just trial-and-error repeated; it is a guided co-evolution where human judgment at each step (setting of validation criteria, interpretation of results, decisions on model refinement) dictates the direction of the search space and the ultimate purported invention.

Human judgment operates by establishing validation criteria, interpreting experimental results, determining whether model refinement is warranted, and ultimately identifying the variant that possesses patentable technical significance. This determination should be substantiated by:

(i) Dated validation criteria requirements specifying biological, chemical, or clinical thresholds employed at each cycle;
(ii) Researcher comments on the experimental outputs about the reasons behind that particular result prompting a revision of the model;
(iii) Version control documentation of the generative model identifying how the experimental feedback influenced the parameters, training data, or architecture; and
(iv) Final selection memoranda as to why the final variant met patentable utility and novelty criteria; and.
(v) Sworn statements that these records are a true reflection of the cognitive process of the researcher at the moments of claim-defining, including cross-references to particular elements of the claim.

The 2025 ruling by the Swiss Federal Administrative Court recognized such a dynamic, stating that a human who contributes substantially to the process of AI data treatment, whose outcome is perceived to be a patentable invention, and who seeks to defend the outcome of this process by patent is a patentee as well.^43^ This standard of recognition and appreciation changes the focus of conception to curation, which may be able to support CRISPR-AI workflows with humans being research directors as opposed to technical implementers. However, the Court neither stated any evidentiary standards to show recognition nor considered the issue of iterative integration on joint inventorship analysis.

### I‌II.C. The Tripartite Inventorship Framework

The recognition pathway is executed in a tripartite framework that identifies three analytically distinct modes of human–AI collaboration in CRISPR research, each with particular evidentiary demands. In the conception pathway, scientists need to retain conventional laboratory records of the entire mental conception before the involvement of AI. For the recognition pathway, researchers must satisfy the evidentiary requirements detailed in Section III.B above dated validation criteria, experimental annotations, version control records, final selection memoranda, and sworn statements cross-referenced to claim elements. No additional evidentiary burden is imposed; the requirements are identical whether the invention is analyzed under the recognition pathway in isolation or as part of the tripartite framework.

The integration pathway acknowledges that AI-assisted invention is often the result of an iterative collaboration between multiple researchers and artificial intelligence systems, rather than the sole conception of one individual. In Pannu v. Iolab Corp., each researcher must contribute significantly to the conception of at least one aspect of the claim. Such contributions can be made at different stages of the iterative process of CRISPR-AI workflows (including problem framing, validation threshold setting, interpretation of unexpected results), as long as they are contemporaneously recorded, materially related to the claimed invention, and reflect claim-defining technical judgment. Collaborative records should let patent examiners determine each researcher’s contribution to the conception.

**Conception pathway (traditional):** Before AI is involved, the human researcher develops a clear and irreversible concept of the entire invention, the particular Cas9 variant with specified modifications, the precise gRNA sequence, the therapeutic use, and AI is only an implementation tool. The evidentiary requirement consists of laboratory records of the full mental conception prior to AI assistance, which can be dated to pre-AI human design. No doctrinal innovation is needed on this path; the traditional analysis of inventorship is applicable.

**Recognition pathway (novel):** The human researcher sets a therapeutic objective and fitness criterion, uses generative AI to search the variant space, and uses claim-defining technical judgment to determine which AI-generated outputs have unexpected technical impacts that meet patentability criteria. The AI produces solutions that are not within the human-conceived solution space; the human

(a) perceives patentable value by domain-specific evaluation,

(b) demonstrates that value by experimental demonstration that produces reproducible technical effects, and

(c) incorporates the output into a concrete conception of the purported invention through refinement.

Evidentiary requirements: As specified in Section III.B, the researcher must contemporaneously document (i) dated validation criteria at each cycle, (ii) experimental annotations explaining model revisions, (iii) version control records showing feedback-driven parameter changes, (iv) final selection memoranda justifying patentable utility and novelty, and (v) sworn statements cross-referenced to claim elements.^44^

The pathway reconceptualizes inventorship by recognizing post hoc identification of patentable technical effects.

**Integration pathway (iterative):** Several human researchers work with AI in a feedback loop where neither a human nor AI conceives the invention independently. One scientist formulates the issue and manages data; another establishes validation standards and analyzes experimental outcomes; the AI creates and optimizes suggestions according to the feedback of the experiment. Conception is shared throughout the human–AI system; invention is created through co-creation. This pathway demands reconceptualized joint inventorship analysis for human–AI collaboration.

The objective standard of non-obviousness in patent law is the person having ordinary skill in the art (PHOSITA) under 35 U.S.C. § 103. The PHOSITA has the skill and knowledge of one of ordinary skill in the art in question at the time of invention. The emergence of AI poses three analytically different issues that are not sufficiently addressed by the existing doctrine. Each CRISPR-AI project can cross all three phases such as problem framing (conception), generative search with experimental validation (recognition), multi-researcher collaboration with refined model (integration). The correct structure varies according to what human input was claim-defining—what decisions determined the particular technical characteristics that were eventually patented.

### I‌II.D. Doctrinal Foundations and Constraints

The recognition pathway maintains the fundamental promises of patent law and adjusts to the realities of computational biotechnology. The framework is influenced by three doctrinal constraints. Natural-person baseline: Inventorship is restricted to natural persons who can comprehend, defend, and be legally responsible over the invention. Under the existing law, AI systems are not permitted to be inventors in every major venue; the recognition channel does not upset this settlement.^45^

Conception as touchstone: The doctrine of the US is that conception is the touchstone of inventorship, the coming into existence of a fixed and irreversible idea of the entire and effective invention. The pathway of recognition is re-conceived to understand how and when conception takes place, but not whether it is necessary. The point at which experimental validation solidifies a clear and irreversible notion of the technical characteristics of an AI-generated variant is recognition.

Significant contribution standard: Under *Pannu v Iolab*, joint inventorship entails a contribution to at least one claim of more than well-known function or direction of the art.^46^ The 2025 Regents decision made it clear, as well, that conception involves the idea and the plan to bring it to practice, and that knowledge that the invention will work in a certain environment is reduction to practice and not conception. CRISPR-AI has all of these requirements.^47^ All these requirements are exerted to the full on CRISPR-AI: recognition should be associated with particular elements of claims, recorded at the time of experimental validation, and not recreated after the fact.

These constraints are met by the recognition pathway. Recognition is a human mental process and an intellectual realization of technical effects carried out by natural individuals. It solidifies conception by means of experimental confirmation, making AI-generated possibilities certain and irreversible inventions. And it needs a claim-specific contribution, which is shown by organized documentation of the human validation decisions regarding the technical features eventually claimed.

The Recognition Pathway can be implemented within the existing structure of U.S. patent law, without the need for an immediate statutory amendment. First, the revised USPTO guidance should make clear that meaningful human contribution in AI-assisted inventions may include claim-defining technical judgment exercised through iterative validation and refinement. Second, standardized evidentiary guidance using the Computational Traceability Report (CTR) and Human-Machine Contribution Statement (HMCS) would ensure the consistency of the documentation of human contribution in an examination. Legislative change would be necessary only if future artificial intelligence systems reach the stage of fully autonomous invention without meaningful human involvement. In the meantime, the Recognition Pathway is moving ahead under the current doctrinal framework articulated by Thaler, Pannu, and the USPTO Inventorship Guidance.

### I‌II.E. Distinguishing Recognition from Directability

The recognition pathway goes further than the augmented inventorship of Levy by pinpointing a specific cognitive point in CRISPR-AI processes.^48^ Directability demands ex ante steering, problem framing, data curation, threshold setting, prior to generative search. Ex post judgment, which is captured by recognition, includes experimental validation, unexpected result discovery, technical effect assessment, once AI has produced solutions.

This distinction is critical to the iterative dynamics of CRISPR-AI. Directability is employed at the beginning of a series of six cycles in the workflow: the principal investigator formulates binding-site goals, rules out cross-reactive epitopes when refining data, establishes early rejection thresholds.^49^ Recognition on an individual cycle: the variants that warrant further investigation are identified, the experimental outcome that has brought to light unforeseen PAM specificity is identified, the conclusion that a given candidate meets therapeutic needs is made. Both are human intellectual products; both can be claim-defining; but they are temporally and cognitively different.

CRISPR-AI does not have sufficient directability without recognition. When a researcher poses the problem, and induces AI production, but imposes no selection on the outputs or interpretation of experimental outputs, the researcher does not invent, despite however complex the problem’s state may be. Recognition may exist even in the absence of directability: a researcher inheriting a generative model, who applies it to produce outputs without parameter modification, but uses qualitative judgment about which outputs should have surprising technical consequences, may qualify as an inventor under the Recognition Pathway.^50^

The Recognition Pathway therefore locates sufficient human control in the exercise of claim-defining technical judgment rather than in the mere operation or supervision of AI systems. Human contribution is legally sufficient where the researcher materially contributes to the conception of the claimed invention by defining scientific objectives, selecting non-routine experimental parameters, establishing validation criteria, interpreting unexpected experimental results, or determining which AI-generated outputs warrant further investigation. By contrast, routine data entry, passive acceptance of AI-generated outputs, or the mechanical implementation of computational recommendations without substantive scientific judgment should not satisfy the conception requirement for inventorship.

The frameworks are not in conflict but are complementary. Augmented inventorship's directability and traceability criteria supply the evidentiary infrastructure for documenting recognition. Ex post recognition under specific evidentiary categories should be documented in the CTR and HMCS, as proposed by Levy:

(i) Validation criteria logs, which capture the thresholds, parameters, and biological assays used at each iteration;
(ii) Experimental results archives, which capture the raw and processed data at each iteration;
(iii) Unexpected findings documentation, which records the unexpected results and their patentable value; and
(iv) Patent office evaluation standards, which confirm that human judgments at claim-defining points are contemporaneously recorded, reproducible, and materially related to the ultimate claimed invention.

## IV. RECONCEIVING PHOSITA FOR CRISPR-AI

The recognition pathway deals with the issue of inventorship attribution; non-obviousness is unresolved. The 2025 USPTO Guidance does not explicitly address the issue of obviousness, stating that, ‘patentability shall not be negated by the manner in which the invention was made’ yet acknowledging that ‘similar questions... may arise under 35 U.S.C. § 103’. This Part develops a five-factor test of non-obviousness in AI-generated CRISPR inventions, dismissing both rigid pre-AI baselines and the computer-skilled-in-the-art (CHOSITA) proposal.

### IV.A. The PHOSITA Problem in Computational Biotechnology

The PHOSITA is patent law's objective standard for non-obviousness. In section 103, an invention cannot be patented when, ‘the differences between the subject matter sought to be patented and the prior art are such that the subject matter as a whole would have been obvious... to a person having ordinary skill in the art’.^51^ The PHOSITA possesses ‘the skill and knowledge of one of ordinary skill in the relevant art at the time of invention’.^52^

The introduction of AI makes this analysis more difficult. Three analytically distinct problems arise. First, the AI-augmented PHOSITA: is it assumed that the skilled individual has access to AI tools as of the filing date? Second, the AI-as-PHOSITA issue: should AI capabilities re-establish a minimum skill level? Third, the obviousness of AI-generated solutions: to what extent does legal manifestness make the solution offered by AI obvious.

The AI-augmented PHOSITA is least controversial. The standard laboratory equipment and knowledge of prior art are assumed in established doctrine. Following KSR International Co v Teleflex, the Supreme Court pointed out that, ‘the results of ordinary innovation are not the subject of exclusive rights under the patent laws’.^53^ In biotechnology, In re Kubin elaborated this method, believing that where the prior art educates a specific enabling methodology and there is a reasonable expectation of success, it may be that the invention is legally obvious even in the unpredictable arts.

By 2025, AI tools such as AlphaFold for protein structure prediction and CRISPR-GPT for gRNA design had become common in computational biology. The AI-enhanced PHOSITA is therefore assumed to have access to such tools; their routine use, without further technical innovation, does not by itself constitute a patentable invention. In Recentive Analytics, Inc. v. Fox Corp. (2025), the Federal Circuit held that ‘simply applying generic and off-the-shelf AI-based models . . . will not pass the Alice § 101 test’, implying a parallel with obviousness under § 103.^54^

Ryan Abbott suggests that the PHOSITA be replaced with a computer with ordinary skill in the art (CHOSITA), based on the idea that artificial intelligence capable of producing inventions worthy of a patent will prompt a shift in the level of obviousness to match that of a machine. Abbott indicates that intelligent machines would be able to gauge intuition in parallel, which gets more reliable through a reproducibility test. This proposal raises four doctrinal issues:

First, CHOSITA mixes the computational ability with legal ability. AI has the capability to completely search sequence spaces and discover patterns in high-dimensional data, but not the judgment, experience, and tacit knowledge that comprise the art of skill.^55^ The PHOSITA is a legal fiction that is intended to measure human innovation; replacing machine capacity would cut the standard off at its epistemological base.

Second, CHOSITA would invalidate human-made inventions in a systematic manner, and AI-generated inventions would be in a patentability dead zone. In Thaler, inventions made by AI and not by humans cannot be patented.^56^ If CHOSITA makes all AI-realizable outputs self-evident, there would be no patent protection on a wide range of technically useful innovations.

Third, CHOSITA disregards the KSR focus on 'ordinary innovation' as the bottom of non-patentability. The PHOSITA is not just a prediction engine but a normative object that defends the public domain against regular progressions; CHOSITA would undermine this object of normalization to pure power of computation.

Fourth, CHOSITA conflicts with the 2025 USPTO Guidance that explicitly considers AI as a tool similar to laboratory equipment, computer software, or research databases but not as an actor in the law that can have a skill. But this position cannot be maintained as AI capabilities evolve.

### IV.B. The Five-Factor Test

This Article advances a five-factor test for the obviousness of AI-generated CRISPR inventions. The framework humanizes PHOSITA, and the role of AI as a predictability indicator becomes legible.

**Factor 1: AI System Architecture Specificity.** This factor determines the generality or problem-specificity of the AI system used. Generic architecture refers to off-the-shelf models (eg, untrained AlphaFold, general-purpose protein design algorithms) used to solve the CRISPR problem without task-specific training, architectural adaptation, or hyperparameter optimization. Problem-specific architecture refers to models that have been optimized on CRISPR-relevant datasets, customized using custom architectures, or trained on proprietary sequence-function data.^57^ The more problem-specific the AI architecture, the less predictable the output and the stronger the indication of non-obviousness.

*Application:* In *Scenario 1* (obvious), a researcher runs unmodified AlphaFold to predict the structure of a known CRISPR target and picks the most confident prediction. Factor 1 is biased toward obviousness since the AI is generic and the application is routine. In Scenario 2 (non-obvious), a team designs a custom variational autoencoder that is trained on rare disease mutations and evolutionary sequence data to design a base editor for an undruggable target. Factor 1 is biased toward non-obviousness since the AI architecture itself is a creative work.

**Factor 2: Problem Structure.** This factor evaluates the research problem as defined or ill-posed. A defined problem has: (a) constraints that are defined (eg, optimize gRNA on a known target sequence with specificity thresholds); (b) a solution space that is finite and searchable; (c) gRNA inputs and outputs that are deterministically or probabilistically coupled. The characteristics of an ill-posed problem are: (i) imprecise or unfamiliarizable constraints (like: design a novel base editor for an unknown therapeutic target), (ii) unbound or dynamically expanding solution space, and (iii) no agreed way of assessing the quality of solutions.

Well-defined problems generally weigh in favor of obviousness, since AI has exhaustive search capabilities, and solutions are predictable; ill-posed problems are biased toward non-obviousness, since human judgment is needed to conceptualize the search space and validation criteria.^58^

*Application:* Optimizing gRNA for a known target with specificity constraints (well-defined) is predictable since the solution space is finite and the success criteria are measurable. The non-obviousness is more probable because designing a base editor for a target with no known binding sites, where the therapeutic mechanism is not well understood, and where off-target effects cannot be predicted ab initio (ill-posed) requires human intuition to structure the problem.^59^

**Factor 3: Solution Space Character.** Finite and searchable versus unbounded. In cases where AI is searching a parameter space of known Cas variants to identify an optimal combination, the solution is predictable. In cases where AI generates a surprising output that is not within the expected solution space, a Cas variant with unexpected PAM selectivity or an off-target profile not predicted by prior art, the output is a true discovery, rather than an optimization.

**Factor 4: Human Judgment Quality.** Identification of the problem, definition of constraints, interpretation of unexpected results. No creative burden is placed on unthinking data entry and automated execution of functions, such as feeding sequences into AI and recording the results. The human judgment that patent law rewards includes: identifying the problem that AI should be applied to; setting success metrics and validation criteria; and determining when AI output indicates unforeseen technical impacts.^60^ This aspect assesses the use of the recognition criterion of Part III by human researchers, or simply the ratification of AI proposals.

**Factor 5: Prior Art Integration.** Direct incentive to implement AI versus new application. In cases where previous art directly instructs the use of AI for the particular CRISPR problem, such as published methods of AI-optimized gRNA design, the skilled practitioner would be guided to the solution. In areas where the field had not previously considered the problem to be solvable by computation, or where the AI application is counterintuitive, the output exhibits creative problem recognition.

### IV.C. Applying the Framework: Illustrative Scenarios

**Scenario 1:** Generic AI, problem is well-defined; space is finite; human judgment is low; there is prior art. A scientist takes the standard AlphaFold unmodified to model protein structures of known CRISPR targets and chooses the most confident prediction. The reasons are 1–5 which all point to obviousness; no patentable invention.

**Scenario 2:** Custom AI, ill-posed problem, unbounded space, high human judgment, novel application. A research team creates a highly sensitive generative model, which trains on rare disease mutations and uses it to design a base editor to an earlier undruggable target, and uses iterative experimental validation to discover a variant with a surprising level of efficiency and specificity, not proposed by previous art. Factors 1–5 indicate non-obviousness; patentable invention.

**Scenario 3:** Mixed factors CRISPR-GPT workflow. A principal investigator establishes therapeutic goals for sickle cell disease, curates training data by omitting cross-reactive epitopes, sets specificity thresholds, and validates AI-generated variants in six iterative cycles, and Factors 1 and 2 (AI system architecture and problem structure) weigh toward non-obviousness. Factors 3–5 require a fact-specific assessment of whether the unexpected PAM specificity, the quality of human technical judgment, and the absence of prior-art guidance collectively support a finding of non-obviousness.

### IV.D. Temporal Baseline: The Moving PHOSITA Problem

The capabilities of AI evolve rapidly, whereas the PHOSITA inquiry is fixed at the time of invention. Accordingly, the appropriate baseline for CRISPR-AI inventions is the state of AI capabilities at the time of the first practical reduction to practice. Technology that required custom AI development in 2023 may become achievable using off-the-shelf tools by 2026.^61^ This approach preserves KSR's prohibition against patenting ordinary technological progress while avoiding hindsight based on subsequent advances in AI.

### IV.E. Distinguishing from CHOSITA and Pre-AI Baselines

The five-factor test occupies a middle ground between the traditional pre-AI PHOSITA baseline and CHOSITA (replacing human skill with machine capacity).^62^ The PHOSITA is also human with AI tools as a standard equipment, but rated on human judgment criteria, such as problem recognition, unexpected result identification, technical effect evaluation, which AI systems are unable to accomplish.

AI capabilities serve as indicators of predictability, not as the baseline for legal skill.^63^ In the case of a finite, well-defined solution space, where AI explores the solution space exhaustively, the answer is predictable and obvious. The outcome can be unobvious where AI produces surprising solutions in ill-posed spaces that human judgment must verify and improve. The question here is human-centered: would an expert human being, endowed with the tools of available AI, and knowledge of the field, have considered the invention as obvious?

## V. COMPARATIVE JURISDICTIONAL MODELS AND HARMONIZATION IMPERATIVES

The recognition pathway and the five-factor obviousness test are working in a fragmented international environment.^64^ With the globalization of CRISPR-AI research, national differences in inventorship laws generate legal ambiguity, perverse incentives in innovation, and forum shopping.^65^ This Part critically assesses four emerging jurisdictional models of AI-assisted inventorship, strict conception (United States), recognition (Switzerland), legislative reform (Japan), and the administrative implementation of the natural-person inventorship rule (China) and states that harmonization via WIPO coordination is the key to computational biotechnology.^66^

### V.A. The United States: Strict Conception and Its Discontents

The 2025 USPTO Revised Inventorship Guidance represents a retrenchment. The Guidance, which rescinds the effort of the February 2024 Guidance to apply the Pannu factors to AI-assisted invention, states that, ‘the same legal standard of determining inventorship should be applied in all inventions, whether or not AI systems were utilized’. The touchstone is still conception in the mind of the inventor where a certain and fixed idea of the entire and functioning invention is formed. This stringent conception criterion has difficulty with the iterative nature of CRISPR-AI. The Guidance acknowledges that conception requires the contribution of one or more natural persons, but it does not provide doctrinal instruments to assess distributed human–AI processes where the crystallization of the invention is experimental validation.

The situation is further exacerbated by the cases of Hess^67^ and Regents^68^: Hess stated that joint inventorship had to be contributed to conception, not just verified; Regents stated that conception had to be accompanied by an idea and a plan of reducing it to practice. The temporal distance between original thought and the final conception can have an impact on joint inventorship analysis with six-cycle CRISPR-AI workflows, in which the human perceives unexpected AI output days or weeks after it has been generated.

The result is a significant compliance dilemma. The USPTO Guidance instructs that ‘inventors who utilize AI tools should make sure that they are contributing enough to the invention conception to be considered inventors’, but does not give any criteria to assess the sufficiency of contribution in iterative workflows. The researchers have to record human input without understanding what records will please the examiners or courts, which adds to prosecution expenses and the risk of litigation.^69^

More importantly, the stringent standard of conception can make valuable CRISPR-AI inventions ineligible for patents. In cases where AI produces a variant with unforeseen PAM specificity that human researchers discover via experimental validation, the definite and permanent idea was created by the AI, not the human. The human contribution is recognition, not conception. The 2025 Guidance will push CRISPR-AI innovation into trade secrecy or, worse, into jurisdictions with more lenient standards by not acknowledging recognition as a valid inventorship pathway.

### V.B. Switzerland: The Recognition Standard and Its Limits

An alternative is provided by the decision of the Swiss Federal Administrative Court in the DABUS case in June 2025.^70^ Affirming that AI cannot be an inventor, the Court held that, ‘a human who makes a significant contribution to the AI data treatment process, perceives its result as a patentable invention, and seeks to protect it by patent is also an inventor’.^71^ This standard of ‘recognition’ and ‘appreciation’ specifically attributes a post-hoc identification of patentable value in AI output.

The Swiss model is more compatible with the iterative nature of CRISPR-AI. The Court stressed that the path to an invention is not final, but the result is, as it is possible to patent an invention that was made by accident or AI, as long as humans see its significance. This is in line with the recognition pathway’s emphasis on experimental validation and the identification of unexpected results.

But the flexibility of the Swiss standard creates uncertainty. The Court did not specify what constitutes a significant contribution to the AI data treatment process or how recognition of patentable value should be demonstrated.^72^ Does curating training data suffice? Setting validation thresholds? Interpreting experimental results? In the absence of formalized standards, the recognition criterion will tend to degenerate into either subjective hindsight or, on the other hand, necessitate such a high level of human intervention that the generative aspect of AI becomes unnecessary.^73^

Iterative workflows have an evidentiary gap that is acute. The DABUS case featured one AI output; CRISPR-AI features hundreds of variants, numerous experimental cycles, and distributed human input.^74^ What should Swiss courts do with recognition in cases where six researchers make contributions to various cycles, or where the key investigator who formulated the problem never personally approved the final form? The Swiss standard leads to the recognition pathway, but needs to take into account the tripartite framework and evidentiary instruments that have been advanced in Part III to be administrable.^75^

### V.C. Japan: Legislative Reform and Strategic Transition

Japan is a two-tailed landscape. The Intellectual Property High Court's January 2025 decision rejected DABUS, holding that ‘the current patent law requires a human inventor’.^76^ However, the Intellectual Property Strategic Program 2025 of the Cabinet Office, published at the same time, suggested immediate legislative action and pledged Japan to actively build international frameworks to invent AI.^77^

This judicial-policy divide brings about strategic uncertainty. The rigid interpretation of the Court is consistent with the US practice; the reform agenda of the Cabinet Office implies the willingness to adopt recognition-pathway or even sui generis frameworks. In the case of CRISPR-AI applicants, the issue is whether to submit now under dubious standards or wait until the legislation is clarified, which may cost them priority dates in a rapidly developing area.

It is important that the Strategic Program focuses on international systems. Japan is also establishing itself as a harmonization leader, which may be a compromise between the US rigid conception and Swiss recognition strategies. The active construction proposed in the Program implies a readiness to create doctrinal innovations that might guide WIPO coordination. The hybrid approach of Japan, which is now characterized by strict judicial interpretation and which is awaiting legislative reform, can provide a model to jurisdictions that want to maintain legal stability while allowing for adaptive evolution.

### V.D. China: Investment-Driven Divergence and Strategic Risk

China combines a clear natural-person inventorship rule with an increasingly detailed regulatory framework for AI-related patent applications. The 2023 Patent Examination Guidelines, as reinforced by the 2025 amendments, maintain that inventors must be natural persons and expressly prohibit listing artificial intelligence systems as inventors.^78^ Although the inventorship rule is now comparatively clear, the Guidelines provide limited guidance on how to assess human inventive contribution in complex iterative human–AI research workflows such as CRISPR-AI. Published scholarship has yet to systematically examine how Chinese examiners apply these inventorship principles to iterative CRISPR-AI inventions.

The broader policy environment is equally significant. China has the highest number of AI patent applications in the world, with more than half of all AI patent applications.^79^ The government is heavily investing in AI biotechnology, as evidenced by state-funded projects such as PaddleHelix protein structure prediction by Baidu and AI drug discovery programs by Tencent.

Whether China's substantial investment in AI biotechnology has been accompanied by distinctive examination practices concerning AI-assisted inventions remains an empirical question requiring systematic analysis of patent examination practices, published administrative decisions, and future judicial interpretations. The existing literature has not yet determined how state funding affects substantive inventorship standards.^80^

One remaining comparative issue concerns the possibility of divergent international approaches to assessing human contribution in AI-assisted inventions. If Chinese examination practice develops a different interpretation of the degree of human contribution required for AI-assisted inventions than that adopted in the United States or Europe, differences in patent outcomes could create incentives for forum shopping.

Nonetheless, existing indications of this divergence are not widespread; the 2023 Guidelines, as reinforced by the 2025 amendments, establish the current doctrinal baseline, and further practice of examination has not been thoroughly investigated in the peer-reviewed literature. Although neither the Patent Law nor the 2025 Patent Examination Guidelines recognizes AI as an inventor, the 2025 amendments further clarify the examination of AI-related patent applications while preserving the natural-person inventorship requirement.

From a comparative perspective, the principal analytical challenge is not uncertainty regarding China's legal position on AI inventorship, but the limited body of published administrative decisions, examination practices, and judicial interpretations explaining how CNIPA applies the natural-person inventorship requirement to sophisticated AI-assisted inventions. As additional administrative decisions, examination practices, and judicial interpretations become available, they are expected to clarify how China's existing inventorship framework applies to complex AI-assisted inventions such as CRISPR-AI.

Within the framework of their exploration of patent examination in China, Liu and Chen remark that the actual implementation of the Patent Examination Guidelines, particularly in the context of AI-assisted inventions, remains insufficiently documented in the published literature and requires further doctrinal clarification. In the absence of empirical support, arguments concerning strategic benefits are speculative.^81^ Whether the evidentiary framework proposed by the Recognition Pathway would integrate effectively into China's administrative patent examination system remains an empirical question that cannot yet be answered on the basis of current evidence.^82^

### V.E. Critical Evaluation: Fragmentation Costs and Harmonization Imperatives

Three types of costs are imposed on CRISPR-AI innovation by jurisdictional fragmentation. The costs of compliance are increased by the fact that multinational research teams have to meet a variety of divergent documentation requirements: US rigid conception demands contemporaneous claim-specific contributions; Swiss recognition demands perception of patentable value; the Japanese reform path is unpredictable; China applies a clear natural-person inventorship rule, although publicly available examination practice concerning AI-assisted inventorship remains limited.^83^

The possibility of forum shopping creates analytical issues regarding whether the jurisdictional choice could be motivated by the differences in patent standards instead of scientific or clinical excellence. Nevertheless, empirical data on this behavior in CRISPR-AI in particular are scarce; the majority of reported forum shopping is tax or regulatory arbitrage, not variation in inventorship standards. Potential delay costs in innovation arise where the researchers are waiting until there is legal clarity, and this may delay the development of therapeutics for rare diseases where CRISPR-AI could provide custom-made solutions.^84^

The need for harmonization is particularly acute in the context of CRISPR-AI. Computational biotechnology, unlike generic AI-assisted invention, depends on experimental validation as a constitutive component of the Recognition Pathway. Those jurisdictions which do not permit recognition risk missing a category of inventions which are:

(i) technically useful,
(ii) socially useful, and
(iii) becoming more and more common in pharmaceutical research.

There are three harmonization mechanisms that are worth considering. WIPO coordination must create a CRISPR-AI-specific working group, separate from generic AI inventorship debates, to come up with non-binding guidelines that capture the experimental validation needs of the field.^85^ Major patent offices (USPTO, JPO, EPO, CNIPA) might agree to bilateral recognition to grant presumptive validity to patents registered under partner-country recognition systems, diminishing forum shopping incentives.^86^ Evidentiary standardization via harmonized documentation requirements—based on the CTR/HMCS instruments of the augmented inventorship framework of Levy—would lower compliance costs without compromising national flexibility on substantive inventorship standards.^87^

The normative stakes are not limited to patent doctrine. CRISPR-AI-based therapies for rare diseases, personalized oncology, and agricultural resilience rely on innovation incentives that are organized by patent law.^88^ A disjointed system whereby inventions in recognition pathways are not patented in key jurisdictions is likely to slow the creation of therapies to patients who have no other options.^89^ Harmonization through the Recognition Pathway does not require identical substantive standards across jurisdictions. Rather, it requires a shared evidentiary framework capable of recognizing meaningful human technical judgment in AI-assisted inventions. Such coordination would advance both innovation policy and public welfare.^90^

## VI. ETHICAL, ACCESS, AND BIOSAFETY IMPLICATIONS

The recognition pathway and five-factor obviousness test are subject to wider ethical and policy limitations that cannot be disregarded by patent law.^91^ CRISPR-AI convergence poses unique biosafety, equitable access, and research accountability challenges that doctrine-based innovation must address.^92^ This Part discusses three imperatives: biosafety disclosure of AI-designed organisms, access provisions for personalized therapies, and data governance for reproducible science.

### VI.A. Biosafety and the Disclosure Function

The disclosure bargain in patent law presupposes that published specifications allow those with the necessary skills to reproduce claimed inventions.^93^ CRISPR-AI contradicts this assumption in two aspects. First, the black box character of certain generative models, especially deep learning models with millions of parameters, may render the process of invention hard to reproduce even when the result is revealed.^94^ Second, AI-created organisms with new PAM specificities or improved editing efficiency can pose biosafety risks that traditional patent examination is poorly positioned to assess.

Recognition pathway exacerbates the initial concern. In cases where human inventors discern in AI output marketable value and lack full knowledge of why the AI should have come up with that specific solution, in the specification can be described as the invention operates but there is no explanation of how it was invented. This is not a case of enablement failure; rather, it is an epistemic opaqueness of machine learning per se.^95^

This is partially addressed by the emphasis of the five-factor test on problem-specific AI architecture (Factor 1) and human judgment quality (Factor 4), which does not forbid the documentation of validation criteria, the identification of unexpected results, but does not eradicate the problem of the opacification of the generative process itself.^96^

CRISPR-AI inventions of novel organismal function should have increased biosafety disclosure requirements.^97^ The CTR/HMCS evidentiary framework suggested by Levy can be modified to incorporate:

(i) AI safety assessments that determine whether the generative model was trained on potentially hazardous sequences;
(ii) containment measures to validate the experiment; and
(iii) environmental risk assessments for use in applications with gene drive or horizontal transfer potential.

Such disclosures would be reviewed by expert biotechnology patent examiners with expertise in computational biology and biosafety, rather than generalist examiners.

The normative justification is not just limited to the conventional disclosure role played by patent law. The ability of CRISPR-AI to produce unexpected biological behavior, such as PAM specificities not found in nature and editing efficiencies larger than those observed by evolution, provides both therapeutic potential and ecological hazards.^98^ The special rights granted by patent law are conditional on benefit to the wider society; where the benefit in this context is indeterminate and the risk is new, increased skepticism is justified.

### VI.B. Access, Equity, and the Personalized Therapy Paradox

The 2024 case of personalized CRISPR therapy, a tailor-made treatment after six months of development for a single infant, is an example of such a therapeutic paradigm that is hard to incentivize by patent law.^99^ Personalized medicine guarantees specialized efficacy; it also divides markets, issues with clinical trials, and the old paradigm of business associated with blockbusters in the mass market.

The implications of the recognition pathway for access are ambivalent. On the one hand, attributing recognition instead of conception can reduce obstacles to patenting personalized therapies and promote investment in AI-based customization. Conversely, when recognition needs a lot of experimental validation, six-cycle iterative workflows with complete documentation, the cost of creating inventorship can become prohibitive by itself in the rare disease applications when the population of patients is small.^100^

The partial solution is the march-in rights in the Bayh-Dole Act.^101^ As for CRISPR-AI therapeutics developed under federal funding, agencies can march in to license competitors in case the patent holder does not put the invention on reasonable terms to the public. The evidentiary requirements of the recognition pathway, which entail recording human judgment in validation and unforeseen outcome discovery, offer objective standards of whether AI development expenditure is worth pricing to make reasonable royalty choices.

More essentially, the recognition pathway implies a reconceptualization of the incentive structure of patents for personalized medicine. Whereas traditional patents are granted based on the conception of universal solutions, recognition-pathway patents are granted based on the infrastructure and expertise to produce custom solutions—AI systems, validation protocols, clinical translation capacity. This changes competitive advantage to not who came up with the best variant of Cas9 but who can most consistently produce patient-specific therapies, an incentive alignment that can be more socially constructive to the social promise of personalized medicine.^102^

### VI.C. Data Governance and Research Reproducibility

The iterative workflows of CRISPR-AI are based on research inputs and even trade secrets, such as genomic sequences, relationships between structure and function, and clinical outcomes.^103^ The evidentiary requirements of the recognition pathway can bring conflict to the fore: recording the human judgment in data curation and validation might need the disclosure of data sources that the applicants do not want to reveal.^104^

Proportional disclosure offers a middle path. Factor 1 (AI system nature) and Factor 4 (human judgment quality) of the five-factor test involve the description of data curation guidelines and validation procedures without necessarily revealing raw sequences or proprietary databases. Factor 5 (prior art integration) will need the AI application to be put in its proper context within the existing literature of methodology, so that trained practitioners can recreate the approach (but not the specific training set). This strikes a balance between transparency and competition protection, recognizing that data advantage is often short lived, with the rise of genomic databases exponentially, and methodological innovation continuing.^105^

These issues are magnified by the reproducibility crisis in AI-driven research. Studies show that machine learning models in biomedicine often cannot be independently replicated because of data leakage, overfitting, or insufficient validation.^106^ This is met by the recognition pathway’s focus on experimental validation as constitutive of invention, not just confirmatory, which demands a physical demonstration of technical effects, not just computational prediction. The AI output that the researcher acknowledges as having patentable value is based on experimental validation, which, in principle, is reproducible.

This can be enhanced through institutional mechanisms. Journal-patent coordination requiring preprint or publication of CRISPR-AI methods as a condition of patent filing would subject claims to peer review and replication attempts before examination. Federally funded research requirements based on data commons, based on NIH genomic data sharing policies, would decrease strategic secrecy and maintain a competitive advantage in methodological innovation.^107^

### VI.D. Synthesis: Ethical Patent Law for Computational Biotechnology

The five-factor obviousness test, recognition pathway, and ethical safeguards created in this Part are a comprehensive CRISPR-AI patent law framework. The framework maintains the fundamental promises of patent law, incentive to innovate, disclosure to the common good, human responsibility to the law, while adjusting to the unique dynamics of computational biotechnology: iterative human-machine processes, epistemic obscurity, personalized therapeutic possibilities, and data-sensitive methodology.^108^

The moral imperatives are not externalities but internal demands of the social contract of patent law. Where AI creates biological functionality that is beyond the imagination of humans, where experimental validation is constitutive of invention, and where personalized therapies disrupt mass-market business models, patent doctrine needs to change to ensure its continued relevance as innovation policy. The recognition pathway provides that evolution: human-focused yet technologically plausible, incentive-sensitive yet access-restrictive, commercially feasible yet ethically limited.

## VII. POTENTIAL OBJECTIONS AND REBUTTALS

The CRISPR-AI inventorship recognition pathway and five-factor obviousness test are vulnerable to a number of criticisms. This Part predicts and addresses six major objections: that recognition smuggles machine personhood by stealth; that it reduces the inventorship bar; that CHOSITA is unavoidable; that international harmonization is impossible; that traceability will kill innovation; and that multi-actor pipelines will degenerate into proprietorship.

### VII.A. De Facto Machine Personhood without Explicit Defense?

The first issue is that by acknowledging the generative nature of AI in the recognition pathway, there is a danger of giving machines legal status indirectly. When solutions generated by AI are credited in the inventive process, does this not personify AI in practice even non-nominally?^109^

The objection misunderstands the structure of the recognition pathway. The doctrine maintains the natural-person threshold that dominant forums have implicated in current tools; inventorship, statements, assignment, and liability accrue to human participants only. AI is merely evidence of the process, its outputs are probative of predictability to analyze obviousness, and legal responsibility is human. The recognition pathway considers AI as a creative partner whose contribution should be demonstrated, whereas legal responsibility is human.

The difference is explained with the comparison of recognition to directability. Where the directability of Levy demands ex ante control of the model behavior, recognition demands ex post evaluation of the effects of the technical impacts. Both are irreducibly human cognitive processes: steering involves problem-framing knowledge; recognition involves domain-specific analysis of the results of the experiment. Both cannot be done by machines; both do not give them legal status.

### VII.B. The Dilution of Inventorship Criteria

A second criticism is the impact of recognition on the incentives to innovate. So, when patenting AI output requires inventors only to recognize it, inventions that do not require much human input can be patented, meaning that the purpose of patent law, namely to incentivize innovation, will be eroded.

The objection mismatches recognition with passive selection. The recognition pathway needs technical effect judgment, or the capacity to determine whether an AI-generated variant can attain therapeutic goals, whether it has binding affinity or off-target cleavage, whether it exhibits unintended behavior outside of model-intended behavior. This is not a trifling input; it is the professional judgment that turns computational possibility into clinical feasibility.

The five-factor obviousness test supports this gatekeeping role.^110^ Factor 4 (quality of human judgment) directly assesses whether the researchers applied problem recognition, constraint definition, and unexpected result interpretation to the problem or simply approved AI suggestions. In the case of human input that is mindless data entry and performance of functions, there is no patentable invention.^111^ The recognition pathway thus maintains incentive structure while adapting to technological reality.

### VII.C. The Question of CHOSITA's Inevitability

The third criticism is that AI capabilities will inevitably redefine the PHOSITA baseline. With AI systems becoming the norm in computational biology, the argument goes, the skilled individual must be assumed to have AI capacity; anything that AI can produce becomes self-evident. This proposal of a computer with common sense in the art (CHOSITA) seems to be technologically predetermined.

The criticism is a misinterpretation of the correlation between ability and talent. AI systems have computational power, exhaustive search of sequence spaces, pattern recognition in high-dimensional data, which are beyond human ability. However, judgment, experience, and tacit knowledge, which cannot be replicated by algorithms, are part of skill in the art. The PHOSITA is a legal fiction that is used to assess human innovation; substituting it with machine capacity would cut the epistemological foundation of patent law.^112^

The five-factor test offers an alternative. Indicators of predictability, rather than skill baseline, are AI capabilities. In cases where AI is exhaustively searching a finite, well-defined solution space (Factors 1–3), the outcome is predictable and obvious; in cases where AI is producing surprising solutions in ill-posed spaces where human judgment is needed to confirm the solution (Factors 4–5), the outcome can be non-obvious. The question is still human-focused: would a human expert, endowed with the available AI tools and knowledge of the field, have considered the invention obvious?^113^ CHOSITA is neither inevitable nor desirable.^114^

### VII.D. Harmonization as an Unattainable Ideal?

A fourth argument is against international coordination. Considering the national interest’s divergence between the US strict conception, the Swiss recognition, the Japanese reform, the Chinese strategic ambiguity, it is politically impossible to harmonize on the recognition pathway.^115^

The objection exaggerates divergence and underestimates convergence pressure. All major jurisdictions have disavowed AI-as-inventor; all demand natural-person inventorship; all are subject to the same technological pressure of CRISPR-AI convergence natural-person inventorship. It is the variation in the valuation of human contribution, not the necessity of such a valuation where WIPO soft-law coordination comes in: form-of-disclosure provisions that modify the manner of demonstrating enablement, but not what is to be demonstrated or by whom one may be an inventor.^116^

The TRIPS framework offers legal grounds. The disclosure provisions of Article 29 and the non-discrimination principle of Article 27 allow for differentiated evidentiary standards for AI-intensive inventions. The bilateral recognition agreements between major offices may have presumptive validity , as discussed in Part V, without necessarily having the same substantive standards.^117^

### VII.E. Discouraging Innovative Development

A fifth concern is that the traceability requirement, a record of judgment by humans at claim-determining points, will also impede innovation by burdening researchers with compliance overhead.^118^

It is more probably the reverse (tie inventorship to reasons which directed model behavior at the claim-element level).^119^ The doctrine minimizes ex ante error costs (clearer drafting; less office action) and ex post litigation costs (cleaner enablement and inventorship records). Records will be short in a place where AI does not influence conception and extensive in a place where it does matter. The recognition pathway provides a grammar that can be administered for accountability where it is most required.

### VII.F. Systemic Breakdown Among Multiple Actors

One last objection is against collaborative research.^120^ In CRISPR-AI pipelines, infrastructure, data, validation, and oversight are provided by multiple researchers; the recognition pathway will be incentive to entrench inventorship rather than management.^121^

The tripartite structure avoids this when multiple researchers each pass the recognition threshold, as discussed in Part III. The question is always: whose reasons fixed which elements of the claim? When multiple researchers each pass the recognition threshold—problem framing, data curation, validation criteria, unexpected result identification—joint inventorship ensues under a normal Pannu analysis.^122^ Where actors provide infrastructure, capital, or overall supervision without influencing conception at claim-defining points,^123^ they are not inventors but can be compensated by contract or institutional means.^124^

## VIII. CONCLUSION

CRISPR-AI convergence requires patent law to reimagine inventorship not only as augmentation, but recognition. It exposes a legal lacuna to the inventorship in iterative methods of human–machine production, where AI comes up with innovations that humans never foresaw, that is not explicitly spelled out by patent law. This ambiguity in doctrine risks putting commercially valuable and therapeutically necessary innovations off limits to the protection of patents, and demands patent law to rethink inventorship not as supplement or enhancement, but as reward.

Computational biotechnology with its iterative feedback loops cannot be represented by the conceptual frameworks of conception, which must have decisive and irreversible concepts held by human minds, prior to the initiation of generative search. The recognition pathway retains human accountability and facilitates technological reality where inventorship is vested in natural persons who exercise intellectual judgment to see patentable value in AI outputs, which are proven through experimental demonstration and refined through trial and error.

The five-factor obviousness test provides an administrable system to assess non-obviousness in AI-enhanced biotechnology. Evaluating the nature of AI systems, the structure of the problem, the character of the solution space, the quality of human judgment, and the integration of prior art humanizes the PHOSITA, and the role of AI can be made legible as a predictability metric. The framework opposes inflexible pre-AI foundations that overlook technological facts and the CHOSITA suggestion that would break the epistemological foundations of patent law.

Immediate threats to innovation incentives are caused by jurisdictional fragmentation. The high conception standard of the United States, the recognition strategy of Switzerland, the judicial-policy bifurcation of Japan, and the strategic ambiguity of China impose compliance costs, forum shopping distortions, and innovation delays that are especially damaging to rare disease research where CRISPR-AI has a custom-made therapeutic potential. WIPO coordination of CRISPR-AI-specific evidentiary standards, not the same substantive rules, but mutually accepted documentation requirements, can maintain national flexibility and minimize transactional friction.

Ethical imperatives are not external demands but internal demands of the social contract of patent law. AI-designed organisms, access to personalized therapies, and data management of reproducible science are exclusive to the benefit of the people. The focus of the recognition pathway on experimental validation as part of invention is an answer to the reproducibility issues of machine learning, whereas the infrastructure-oriented incentive alignment can be more useful to personalized medicine than the standard blockbuster patenting.

Whether the patent law should evolve to accommodate AI-based biotechnology is not the question, but how to do it without compromising the innovation, ethics, and the balance between exclusivity and disclosure. The recognition pathway provides that evolution: human-oriented yet technologically feasible, incentive-sensitive yet access-sensitive, commercially viable yet ethically limited. Courts and patent offices now need to come up with doctrinal tools to assess recognition in computational biotechnology or risk locking out good inventions from protection at the point where they are most required.

## Footnotes

1 Profluent, *Profluent, Profluent Successfully Edits Human Genome with OpenCRISPR-1, the World’s First AI-Created and Open-Source Gene Editor, Business Wire (Apr. 22, 2024),*  https://www.businesswire.com/news/home/20240422399482/en/Profluent-Successfully-Edits-Human-Genome-with-OpenCRISPR-1-the-Worlds-First-AI-Created-and-Open-Source-Gene-Editor (accessed Dec. 20, 2025).

2 Ewen Callaway, *AI Systems May Need Their Own Lawyers, Experts Say*, Nature (July 30, 2025), https://www.nature.com/articles/d41586-025-02135-3 (accessed Dec. 21, 2025).

3 J. A. Ruffolo et al., *Design of Highly Functional Genome Editors by Modelling CRISPR-Cas Sequences*, 645 Nature 518–25 (2025).

4 See S. Russell, *Profluent's AI-Designed Gene Editor Glimpses into Generalizable Platform*, Genet  Eng. & Biotechnology  News (July 30, 2025), https://www.genengnews.com/topics/artificial-intelligence/profluents-ai-designed-gene-editor-glimpses-into-generalizable-platform/ (accessed Dec. 20, 2025).

5 See Ruffolo et al., *supra* note 3 (describing LLM generation of 4 million novel CRISPR-Cas proteins, with experimental validation of OpenCRISPR-1).

6 M. Jinek et al., *A Programmable Dual-RNA-Guided DNA Endonuclease in Adaptive Bacterial Immunity*, 337 Science 816 (2012); J. A. Doudna & E. Charpentier, *The New Frontier of Genome Engineering with CRISPR-Cas9*, 346 Science 1258096 (2014).

7 J. Abramson et al., *Accurate Structure Prediction of Biomolecular Interactions with AlphaFold 3*, 630 Nature 493 (2024).

8 Y. Qu et al., *CRISPR-GPT for Agentic Automation of Gene-Editing Experiments*, Nat. Biomed. Eng. (July 30, 2025) (online publication).

9 See generally M. Khoshandam et al., *CRISPR/Cas and Artificial Intelligence to Improve Precision Medicine: Future Perspectives and Potential Limitations,* 42 Hum. Gene 201356 (2024) (surveying AI-CRISPR integration).

10 Burroughs Wellcome Co. v. Barr Labs., Inc., 40 F.3d 1223, 1227-28 (Fed. Cir. 1994).

11 U.S. Patent & Trademark Office, *Revised Inventorship Guidance for AI-Assisted Inventions*, 90 Fed. Reg. 73983 (Nov. 28, 2025) (Docket No. PTO-P-2025-0014).

12 Thaler v. Vidal, 43 F.4th 1207 (Fed. Cir. 2022), cert. denied, 143 S. Ct. 1783 (2023); Thaler v. Comptroller-Gen. of Patents, Designs & Trade Marks [2023] UKSC 49, ¶¶ 56–59, 99 (U.K.); J 8/20 and J 9/20, Designation of Inventor/DABUS (EPO Legal Bd. App. Dec. 21, 2021); see also Eur. Patent Office, Press Communiqué (July 6, 2022); Swiss Fed. Admin. Ct., Case B-2532/2024 (June 26, 2025) (Switz.); Japan Intell. Prop. High Ct., Judgment of Jan. 30, 2025, Reiwa 6 (2024) Gyo-Ko No. 10006.

13
*This workflow is analyzed in greater detail in Part II.C and serves as the paradigmatic example throughout this Article.*

14 D. Repecka et al., *Expanding Functional Protein Sequence Spaces Using Generative Adversarial Networks*, 3 Nature  Mach. Intell. 356 (2021).

15 See A. C. Comer, *AI: Artificial Inventor or the Real Deal?*, 22 N.C. J.L. & Tech. 447 (2020); J. Villasenor, *Reconceptualizing Conception: Making Room for Artificial Intelligence Inventions*, 39 Santa  Clara  High  Tech. L.J. 197 (2022).

16 See D. L. Schwartz & M. Rogers, *'Inventorless' Inventions? The Constitutional Conundrum of AI-Produced Inventions*, 35 Harv. J.L. & Tech. 531 (2022); N. Selvadurai, *Inventions Without Inventors: The Need to Recognize AI Systems as Inventors*, 16 Case W. Res. J. L. Tech. & Internet 37 (2025).

17 Haim V. Levy, *Augmented Inventorship: Rethinking Patent Law in the Age of Generative AI*, 13 J.L. & Biosciences 1 (2026).

18 See Ryan Abbott, *Everything Is Obvious*, 66 UCLA L. Rev. 2, 2 (2019) (arguing AI-generated inventions should be treated as obvious); Mark A. Lemley & Brian Casey, *Inventing AI*, 112 Calif. L. Rev. 1, 1 (2024) (critiquing CHOSITA and emphasizing human judgment).

19 Luciano A. Marraffini, *CRISPR-Cas Immunity in Prokaryotes*, 526 Nature 55–61 (2015).

20 Doudna & Charpentier, *supra* note 6.

21 Jinek et al., *supra* note 6.

22 Ewen Callaway & Heidi Ledford, *CRISPR: Gene Editing Is Just the Beginning*, 531 Nature 156 (2016).

23 Haydar Frangoul et al., *CRISPR-Cas9 Gene Editing for Sickle Cell Disease and β-Thalassemia*, 384 N. Eng. J. Med. 252 (2021).

24 Emily Waltz, *Gene-Edited CRISPR Mushroom Escapes US Regulation*, 532 Nature 293 (2016).

25 Rodolphe Barrangou & Jennifer A. Doudna, *Applications of CRISPR Technologies in Research and Beyond*, 34 Nature  Biotechnology 933 (2016).

26 See Jacob S. Sherkow, *A ‘Bare Hope of a Result’: The Second CRISPR Patent Appeal*, 8 Crispr J. 317, 317–20 (2025) (analyzing Federal Circuit's May 2025 *Regents* decision); see also Jacob S. Sherkow, *The CRISPR Patent Dispute and Scientific Collaboration*, 531 Nature 156 (2016).

27 See Regents of Univ. of Cal. v. Broad Inst., Inc., No. 2022-1594 (Fed. Cir. May 12, 2025).

28 John Jumper et al., *Highly Accurate Protein Structure Prediction with AlphaFold*, 596 Nature 583 (2021).

29 Abramson et al., *supra* note 7.

30 DeepMind, *AlphaFold DB* , https://alphafold.ebi.ac.uk/ (accessed Apr. 2026) (reporting 300 M+ predicted structures); see also Jumper et al., *supra* note 28.

31 Beam Therapeutics, *Beam Therapeutics Highlights Progress Across Base Editing Portfolio and Outlines 2024 Anticipated Milestones* (GlobeNewswire, Jan. 8, 2024), https://www.globenewswire.com/news-release/2024/01/08/2805214/0/en/Beam-Therapeutics-Highlights-Progress-Across-Base-Editing-Portfolio-and-Outlines-2024-Anticipated-Milestones.html (accessed Mar. 12, 2026).

32 F. Allen et al., *Predicting the Mutations Generated by CRISPR-Cas9*, 9 Nature  Biotechnology 985 (2021).

33 See Ruffolo et al., *supra* note 3 (describing AI-generated protein designs unanticipated by researchers).

34 See generally Qu et al., *supra* note 8.

35 See Thaler, *supra* note 12, 1213 (Fed. Cir. 2022); United States Patent and Trademark Office, *Revised Inventorship Guidance for AI-Assisted Inventions*, 90 Fed. Reg. 73,983 (Nov. 28, 2025) (Docket No. PTO-P-2025-0014).

36 See, eg, Comer, *supra* note 15; Villasenor, *supra* note 15.

37 See Levy, *supra* note 17.

38 See Schwartz & Rogers, *supra* note 16; Selvadurai, *supra* note 16.

39 See Daniel J. Gervais, *Deconstructing AI Inventorship*, 102 J. Pat. & Trademark  Off. Soc'y 123 (2022).

40 See Y. Qu et al., CRISPR-GPT for Agentic Automation of Gene-Editing Experiments, 10 Nat. Biomed. Eng. 245–58 (2026).

41 See Lemley & Casey, *supra* note 18, at 23–25 (critiquing CHOSITA and emphasizing human judgment in skill definition).

42 See KSR Int'l Co. v. Teleflex, Inc., 550 U.S. 398, 421 (2007) (obvious to try requires reasonable expectation of success; biological unpredictability requires human judgment).

43 Swiss Federal Administrative Court, *infra* note 60, at ¶ 4.2.

44 See *supra-Section III.B (items i-v and accompanying text)*

45
*Id.*; Thaler v. Comptroller-General, *supra* note 12; J 8/20 and J 9/20, *supra* note 12.

46 See Pannu v. Iolab Corp., 155 F.3d 1344, 1351 (Fed. Cir. 1998).

47 Regents, *supra* note 27.

48
*Id*.

49 See Levy, *supra* note 17, at text accompanying note 17 (principal investigator example).

50 See Swiss Federal Administrative Court, *infra* note 60.

51 35 U.S.C. § 103 (2018).

52 In re O'Farrell, 853 F.2d 894, 899–900 (Fed. Cir. 1988).

53 KSR, *supra* note 42, at 420–21.

54 Recentive Analytics, Inc. v. Fox Corp., 134 F.4th 1205 (Fed. Cir. 2025).

55 Lemley & Casey, *supra* note 41, at 23–25.

56 See Thaler, *supra* note 12.

57 See Levy, *supra* note 17 (distinguishing generic and problem-specific AI).

58 See KSR, *supra* note 53, at 421 (finite, predictable solutions).

59 See In re Deuel, 51 F.3d 1552, 1559 (Fed. Cir. 1995), abrogated by *Kubin*, 561 F.3d 1351 (structural novelty in unpredictable arts).

60 See Swiss Federal Administrative Court, Case B-2532/2024 (June 26, 2025).

61 See Fiers v. Revelle, 984 F.2d 1164, 1168 (Fed. Cir. 1993) (conception through reduction to practice).

62 See Abbott, *supra* note 18; Lemley & Casey, *supra* note 41.

63 See KSR, *supra* note 53.

64 See generally WIPO, *WIPO Conversation on Intellectual Property and Artificial Intelligence* (2019–24).

65 See Sherkow, *supra* note 26 (CRISPR Journal article, 2025).

66 See TRIPS, arts. 27, 29 (Apr. 15, 1994).

67 Hess v. Advanced Cardiovascular Sys., Inc., 106 F.3d 976 (Fed. Cir. 1997) (conception is touchstone of inventorship; verification alone insufficient for joint inventorship).

68 Regents, *supra* note 27 (clarifying conception standard in CRISPR priority dispute).

69 See Gowling WLG, *Fragmented and Shifting CRISPR Patent Landscape* (Sept. 24, 2025), https://gowlingwlg.com/en/insights-resources/articles/2025/crispr-patent-landscape (accessed Dec. 20, 2025).

70 Swiss Federal Administrative Court, *supra* note 60.

71
*Id* at ¶ 4.2.

72 Swiss Federal Administrative Court, *supra* note 60, at ¶ 4.2.

73 See M. A. Lemley & B. Casey, *Inventing AI*, 112 Calif. L. Rev 1, 34–36 (2024) (critiquing one-size-fits-all approaches).

74 See Y. Qu et al., *supra* note 40.

75 See Lemley & Casey, *supra* note 18, at 34–36 (critiquing one-size-fits-all approaches).

76 Japan Intellectual Property High Court, Judgment of Jan. 30, 2025, Reiwa 6 (2024) Gyo-Ko No. 10006.

77 Japan Cabinet Office, *Intellectual Property Strategic Program 2025* (June 2025).

78 CNIPA, *Patent Examination Guidelines* (2023, as amended 2025), Part II, Ch. 9, § 6.1.2.

79 WIPO, *Technology Trends 2024: Artificial Intelligence* 12–15 (2024).

80 See *Id.* at 89–94.

81 See WIPO, *supra* note 79.

82 See CNIPA, *supra* note 78.

83 See USPTO, *supra* note 11; Swiss Federal Administrative Court, *supra* note 60; Japan Cabinet Office, *supra* note 77; CNIPA, *supra* note 78.

84 See Frangoul et al., *supra* note 23.

85 See WIPO, *supra* note 79.

86 See TRIPS, *supra* note 66, arts. 27, 29.

87 See Levy, *supra* note 17.

88 See Frangoul et al., *supra* note 23; Beam Therapeutics, *Annual Report 2024* (2025).

89 See Frangoul et al., *supra* note 23.

90 See generally WIPO, *supra* note 79.

91 See generally I. Glenn Cohen et al., *The Oxford Handbook of Law and Ethics of AI* (2024).

92 See Jacob S. Sherkow, *Law, History and Lessons in the CRISPR Patent Conflict*, 33 Nature  Biotechnology 256, 256–57 (2015), doi:10.1038/nbt.3160 (discussing ethical dimensions).

93 See 35 U.S.C. § 112(a) (2012) (enablement requirement); Convention on the Grant of European Patents (European Patent Convention), art. 83 (sufficiency of disclosure).

94 See Ziad Obermeyer et al., *Algorithmic Bias in Healthcare*, 366 Science 447 (2019) (discussing opacity in medical AI).

95 See Obermeyer et al., *supra* note 94.

96 See Part IV, text accompanying notes 126–41.

97 See Sherkow, *supra* note 92.

98 See Callaway & Ledford, *supra* note 22.

99 See Frangoul et al., *supra* note 23.

100 See Frangoul et al., *supra* note 23.

101 35 U.S.C. § 203 (2012) (Bayh-Dole Act march-in rights).

102 See Cohen et al., *supra* note 91.

103 See Y. Qu et al., *supra* note 40.

104 See Levy, *supra* note 17.

105 See Qu et al., *supra* note 40.

106 See S. Nature, *Reproducibility Crisis in Machine Learning*, 573 Nature 185 (2019).

107 See NIH, *Genomic Data Sharing Policy* (2014).

108 See Cohen et al., *supra* note 91.

109 See Selvadurai, *supra* note 16.

110 See Part IV, text accompanying notes 126–54.

111 Pannu, *supra* note 46, at 1351.

112
*See supra-Part IV.A (text accompanying notes The rebuttal is summarized here; the full analysis is reserved to the primary treatment in Part IV)*

113 See Graham v. John Deere Co., 383 U.S. 1, 17–18 (1966).

114 See Part IV, text accompanying notes 149–54.

115 Sherkow, *supra* note 92.

116 See TRIPS, *supra* note 66.

117 See WIPO, *supra* note 64.

118 See Gervais, *supra* note 39.

119 See Levy, *supra* note 17.

120 See Pannu, *supra* note 111, at 1351–52.

121 See Gervais, *supra* note 39.

122 See Pannu, *supra* note 46.

123 See Pannu, *supra* note 46.

124 See Part III, text accompanying notes 78–83.

